# Enzyme reference materials: their place in diagnostic enzyrnology

**DOI:** 10.1155/S1463924694000064

**Published:** 1994

**Authors:** D. W. Moss

**Affiliations:** Rqal Postgraduate Medical School Hammersmith Hospital London W12 ONN UK

## Abstract

Estimations of the activities of various enzymes in clinical samples are routine tasks for clinical chemists. Most of this work is done by automatic analysis. The reference ranges against which patients' results are interpreted, however, have generally been defined in terms of manual methods and the conditions of a manual method cannot be reproduced in automated systems. This paper describes the possibility of translating the results of enzyme analysis into a common currency through the use of enzyme reference materials as calibrators.


Journal of Automatic Chemistry, Vol. 16, No. 2 (March-April 1994), pp. 63-66

### Enzyme reference materials: their place in diagnostic enzymology

**D. W. Moss**

*Royal Postgraduate Medical School, Hammersmith Hospital, London W12 0NN*

*Estimations of the activities of various enzymes in clinical samples are routine tasks for clinical chemists. Most of this work is done by automatic analysis. The reference ranges against which patients' results are interpreted, however, have generally been defined in terms of manual methods and the conditions of a manual method cannot be reproduced in automated systems. This paper describes the possibility of translating the results of enzyme analysis into a common currency through the use of enzyme reference materials as calibrators.*

### Introduction

The introduction of automated methods has had a profound effect on the practice of chemical analysis, not merely in the greatly increased productivity that has occurred, but also in the greater reproducibility of results that automated methods can achieve compared with repetitive manual analysis. In clinical chemical analysis, in particular, current workloads and standards of analytical performance could not have been been contemplated without the advent of automatic analysis. However, automated methods frequently introduce constraints which are not present in their manual counterparts. These include limitations on the relative volumes of samples and reagents that can be dispensed, the inability to vary the cycle time of reactions which consequently may not reach equilibrium, and difficulties in making accurate absolute measurements of quantities such as light-absorbance because of restrictions imposed by the geometry of the measuring system. Almost invariably, therefore, automatic analysers depend on comparative, rather than absolute, calibration, in which reference materials (calibrators) of known or assigned analyte concentrations are introduced into each analytical run, the concentrations in the unknown samples being inferred from comparison of their instrumental signals with those of the calibrators.

The constraints, as well as the advantages, of automatic analysis are particularly apparent in adapting methods whose results are numerically method-dependent, as in the quantitative measurement of enzyme activities. The concentration of an enzyme in a given sample is typically measured in terms of its catalytic activity, i.e. its effect on the rate of a specific chemical reaction proceeding under defined conditions. The defined conditions are chosen and optimised in terms of manual analysis. It is often difficult (and sometimes impossible) to translate these defined conditions without modification into a protocol for a particular automatic analyser, although the ability of automatic analysers reproducibly to ensure accurate timing intervals is a great advantage in the rate measurements required in enzyme analysis. Newer developments in simplified analytical systems in which reactions occurring in moist films or layers of reagents have replaced traditional procedures in dilute aqueous solutions have further complicated the translation from manual to automated analysis.

Many thousands of estimations of the activities of various enzymes in clinical samples are made daily throughout the world to aid diagnosis. The majority are made by various types of automatic analysis. However, reference ranges against which patients' results are interpreted have generally been defined in terms of manual methods, the conditions of which cannot be reproduced in automated systems. This paper looks at the possibility of translating the results of enzyme assays into a common currency through the use of enzyme reference materials as calibrators.

### Reference materials

The term 'reference materials' encompasses a range of materials and their applications in clinical chemistry. At the highest level are preparations of substances of known and reproducible purity, with substance concentrations defined in terms of mass, either by weighing in the case of a pure substance or by measurement by a defined reference method. Lower levels in the hierarchy of reference materials are occupied by materials of different degrees of purity presented in different matrices and mixed with other substances, or even with undefined substance concentrations.

Reference materials of particular hierarchical levels are expected to fulfil different functions in analysis: as *controls*, to verify the transferability of specified measurements procedures between laboratories and the comparability of their results, and to monitor the daily performance of routine analysis; and as *calibrators*, to provide standards of known or defined composition, with which the analytical signals provided by unknown samples analysed in the same run are compared and the corresponding results are calculated. While the requirements for a control and a calibrator are often similar, and a single material may potentially be capable of fulfilling either function, no material can occupy both roles simultaneously. A requirement common to all reference materials is that they should have assured stability within a defined period. For many years enzyme materials were thought incapable of fulfilling this requirement. However, most enzymes of diagnostic significance have been shown to retain their activities almost indefinitely under appropriate conditions: the predictions of accelerated degradation tests, confirmed by real-time measurements on stored preparations, show a negligible loss of catalytic

© 1994 IFCC

activity over a period of years, even for enzymes generally regarded as particularly labile (for example prostatic acid phosphatase).

### The problem of method-dependent results

Special problems in the use of reference materials arise when the numerical results of a measurement procedure are method-dependent, as is typically (although not exclusively) the case with measurements of enzymes in biological samples in terms of their catalytic activities. It is not impossible to envisage the assignment of values to enzyme reference preparations in terms of mass, for example, by weighing-in pure enzymes or by extrapolation from active-site titration. However, enzyme reference preparations currently available have catalytic concentrations assigned by one or more measurement procedures; i.e. they offer method-dependent values. The assignment to a given material of a method-dependent value is appropriate when it is intended for the internal control or external assessment of the performance of that defined method. However, problems arise in External Quality Assessment (EQA) in evaluating results obtained by the variety of methods still in use. Such results from large surveys typically fall into several method-groups, each with its consensus mean value about which, in the better-defined groups, individual results are tightly distributed. Can the differences between the means of such groups confidently be assumed to represent method dependent differences between equally valid estimates of the same catalytic activity, or do they conceal a bias (i.e. nonspecificity) on the part of certain methods? Furthermore, when excessive scatter of results is observed within a method-group, does it really represent poor analytical performance, or are the methods constituting that group in fact heterogeneous?

One solution to these dilemmas is to define a single, method-dependent value for the survey specimen. Also, attempts have been made to assess the performance of enzyme analyses by the relationship found between two samples circulated simultaneously; i.e. by a limited calibration exercise [1, 2]. This paper is mainly concerned with the question of whether, or to what extent, the principle of inter-method calibration can be extended to solve the problems of method-dependency of results in clinical enzymology, not only in EQA but also in daily practice.

The approach to the standardization of method-dependent results that has been pursued most vigorously is through the adoption of agreed routine analytical methods. More than 20 years of efforts directed towards the definition of consensus methods, intended to become nationally or internationally accepted routine methods, have met with a good deal of success [1, 2, 3]. They have focused attention on the inadequacies of many previously used methods, and have raised the level of expectation of users with respect to standards of precision and freedom from bias. However, it now seems clear that the goal of a single, universally-used method for measuring the catalytic concentration of a given enzyme will not be achieved, mainly because of the constant pressures of technical improvement. Attempts have been made to extend the currency of recommended methods by widening the definition of experimental parameters to an extent that does not cause the results obtained to vary by more than 5% from those of the unmodified method [4]. However, such relaxations cannot take account of major changes in procedures, such as the use of different substrates, or 'dry chemistry' techniques.

### Enzyme reference materials as calibrators

The use, as calibrators, of enzyme reference materials of assured stability, and with catalytic concentrations assigned by an agreed reference method, offers the possibility of reporting the catalytic concentrations of enzymes in patients' samples in terms of the units defined by the reference method, even though that method was not in fact used. Thus, a method can be chosen that is more suited to the laboratory's resources or to the demands of routine work than the reference method. However, before such a procedure can be embarked upon, certain criteria must be satisfied relating to the selection both of the calibration material and of the reference and routine methods between which values are to be transferred. Moreover, when choices of calibrator and methods have been made, their validity must be subjected to rigorous experimental verification. These criteria can be subsumed under the general heading of 'commutability' between methods. The concept of commutability has been variously defined. However, for the present purposes, it can be defined as the existence of an identical and constant numerical relationship, within the limits of experimental error, between the results given by two analytical methods for all samples, including both the calibrator and patients' samples [5].

### Essential criteria in the use of enzyme calibrators

*The chosen routine and reference methods must have identical, or at least closely similar, specificities for the analyte.* Where the analyte is an enzyme this requires, among more general factors, similar selectivities towards individual isoenzymes or isoforms. For example, commutability should not be expected between two methods for measuring the catalytic concentrations of acid phosphatase in serum that have differing selectivities for prostatic and non-prostatic isoenzymes. Similarly, methods for assay of aminotransferases that differ in the presence or absence of pyridoxal-5'-phosphate are non-commutable, since they measure different analytes. The chosen methods should be equally sensitive to sample-dependent, enzymatic or non-enzymatic side-reactions.

It is important to recognize that the performance characteristics of the reference method, such as precision or absence of bias, are not transferred to the routine method by the use of a calibrator: the analytical quality of the calibrated results is determined by the characteristics of the routine method itself. However, the use of a within-batch calibrator as a basis of calculation is often observed to improve between-batch reproducibility of results, compared with calculation on the basis of pre-selected parameters such as the absorption coefficient of a reaction product. This occurs when small, between-batch variations in reaction conditions (for example, instrumental settings and timing) affect both patients' samples and the calibrator to exactly the same extent. (Of course, this effect of a calibrator in no way removes the need for the independent control specimens that are equally essential whatever method of calculation is used.)

The methods used to assign catalytic concentrations to enzyme reference materials are typically high-level methods, designed to eliminate as far as possible all known causes of bias, i.e. to ensure that the method is as specific as possible for the analyte enzyme. Furthermore, newer methods usually benefit from advances in the theory and practice of defining reference values. It cannot be automatically expected, therefore, that if the reference method and the routine method are applied in parallel to a series of patients' specimens, the same specimens will be identified as abnormal by each method.

If the routine method is calibrated in terms of the reference method by means of an enzyme calibrator, and if the reference interval of the routine method has been translated into a reference interval for the recalibrated results by application of the factor that expresses the relationship between the two methods found for the calibrator, the classification of the recalibrated results will be no better and no worse than that obtained with the uncalibrated routine method. If, however, the inter-method ratio of the upper (or lower) reference limits is not the same as that of the calibrator, different classification of the specimens will result if the recalibrated results are interpreted against the original reference interval of the reference method.

The possibility of different specimen-classifications can be much reduced by an appropriate choice of calibrators and of routine and reference methods, and, above all, by an extended experimental trial in which catalytic concentrations directly measured by the reference method are compared with those given by the calibrated routine method for a large number of patients' specimens.

*A second essential is that the properties of the enzyme calibrator should be as similar as possible to those typical of the analyte enzyme in its natural matrix, usually human serum or plasma.* In other words, the numerical ratio of the catalytic concentrations determined by the routine and reference methods for the calibrator must be the same as the average ratio found for a large number of patients' samples.

In principle, this is most easily achieved by adding the relevant human (iso)enzyme to a human serum or plasma pool. In practice, the supply of human enzymes may be restricted by ethical and hygienic considerations, while the presence of a basal level of the analyte (iso)enzyme and other uncontrolled reactivities are further complications in selecting a serum or plasma matrix. However, enzymes from animal tissues or from genetically engineered cells can closely mimic the properties of their human analogues, and, since the protein content and ionic composition seem to be the most important features of the enzyme-containing matrix, the use of appropriately chosen non-human enzymes and synthetic matrices does not invalidate enzyme calibration materials in many cases.

*The third criterion for successful calibration of a routine method in terms of a reference method is that the numerical ratio of results obtained by the two methods should be constant (within the limits of experimental error) for every patients' sample.* No unequivocal guarantee can be given that this will be so. However, the probability that a sample-independent ratio exists is increased by careful choices of methods and calibrators, and can be further increased by extending the number and range of samples for which the ratio is experimentally determined by the two methods.

The acceptable variation of the inter-method ratio that is considered to be within the limits of experimental error and therefore consistent with the absence of a sample-dependent variation depends on the characteristics of the routine and reference methods. Some variation in the slope of the regression line relating results given by the routine method to those obtained by the reference method when applied to the same samples is inevitable, because each method has its own inherent imprecision. To determine whether the distribution of experimental points about the regression line arises solely from the imprecision of the respective methods, or whether there is, in addition, a sample-dependent variation (the requirement for commutability is not met) is not a simple statistical problem. Statistical techniques for predicting the variation of the inter-method due to the known imprecisions of the respective methods are available [6]. These can be used to compare the predicted sample-independent, variation of the inter-method ratio with that observed when a sufficiently large number of samples are analysed by each of the two methods. An alternative pragmatic approach is to determine, in such a series of samples, that some arbitrarily-chosen limit of variation is not exceeded. For example, a coefficient of variation of the inter-method ratio of the order of $\pm 2{\cdot}5\%$ about the mean for a large number of patients' samples has been suggested, but this may be unnecessarily rigorous for some methods and applications [6].

The process of establishing that an acceptable degree of commutability exists between a routine method and a reference method thus requires a combination of theory and practice: first, the choice of methods similar in analytical principles, and, above all, in specificity for the analyte to be determined; second, an experimental demonstration of the similarity of their relative response in the two methods for both the calibration material and a number of patients' samples that is large enough to give a significant probability that subsequent samples will conform to the observed ratio. Ideally, the number of samples would be infinitely large. In practice, the number will be at least 50, and preferably 100 or more, covering a wide range of catalytic concentration.

Detailed recommendations for such a study have been proposed [7] and have been applied in a number of studies [5]. These have shown that calibration of one method in terms of another is feasible, provided that calibration materials and measurement procedures are carefully chosen. In several such studies the enzyme calibration materials prepared for the Community Bureau of Reference (BCR) of the European Commission have been used: although the primary and certified use of these materials is to provide a standard with a defined catalytic concentration when measured in a closely specified measurement procedure, data have been provided that demonstrate the near-identity of the catalytic properties of enzyme preparations from animal sources with the homologous enzymes in human serum, for example, gamma-glutamyltransferase from pig kidney [8].

Some of these preparations have also been used to test the principles of commutability between methods set out above. Use of the BCR pig-kidney $\gamma$-glutamyltransferase preparation (CRM 319) as a calibrator showed excellent commutability between the Scandinavian recommended method and the IFCC's reference method, although the methods differ in the nature of the donor substrate and in measuring temperature [5]. Similarly, when the BCR pig-kidney alkaline phosphatase, preparation (CRM 371) was used to calibrate the Scandinavian recommended method in terms of the IFCC provisionally defined reference procedure, commutability was found to be good, with an inter-method ratio for the calibrator matching that found for patients' samples [5]. This good commutability demonstrates that the specificities of the two alkaline phosphatase methods are the same: i.e. that the IFCC method, with its addition of a controlled concentration of zinc ions, does not reveal the presence of zinc-deficient apo-alkaline phosphatase in human serum that is not detected by methods in which zinc is not added. The value of zinc in the IFCC method is thus to reverse, or prevent, any inactivation of the pre-existing alkaline phosphatase caused by impurities in the aminopropanol buffer, rather than to activate zinc-deficient phosphatase. If a significant population of reactivatable, zinc-deficient alkaline phosphatase molecules were present in serum or plasma, the proportion of such molecules expressed as a fraction of the total alkaline phosphatase might have been expected to show a sample-dependent variation; as indeed is the case for the relative proportions of apo- and holo-aminotransferases.

### The way forward

The method-dependence of results of determinations of the catalytic concentrations of enzymes continues to present problems in clinical enzymology, not only in the comparison of results of individual patients investigated in different laboratories, but also in the assessment of EQA surveys. In spite of its undoubted successes, the recommended method approach seems unlikely to make further contributions to the solution of this problem. The time appears to be ripe for exploration of the use of enzyme reference materials as inter-method calibrators: an exploration that should engage the same levels of collaborative effort and experimental rigour that were deployed in support of the recommended-method approach.

### References

1. Jansen, R. T. P. and Jansen, A. P., *Annals of Clinical Biochemistry*, **20** (1983), 52.
2. Bullock, D. G., Moss, D. W. and Whitehead, T. P., *Annals of Clinical Biochemistry*, **23** (1986), 577.
3. Horder, M., Gerhardt, W., Karkonen, M., Magid, E., Pitkanen, E., Stromme, J. H., Theordorsen, L. and Waldenstrom, J. *Scandinavian Journal of Clinical Laboratory Investigation*, **41** (1981), 107.
4. Gerhardt, W., *ECCLS Document Number* 3–4 (Lund, 1988).
5. Moss, D. W., *The 6th International Symposium on Quality Control, Osaka* (Excerpta medica, Tokyo, 1988).
6. Armitage, P., *Statistical Methods in Medical Research* (Blackwell, Oxford, 1971).
7. Moss, D. W. *ECCLS Document Number 5* (Lund, 1988).
8. Schiele, F., Siest, G., Moss, D. W. and Colinet, E., *The Certification of the Catalytic Concentration of Gamma-glutamyltransferase in a Reconstituted Lyophilized Material.* (CRM 319, Community Bureau of Reference, Report EUR 10628 EN, CEC, Luxembourg, 1986).

